# A holistic review of the medical school admission process: examining correlates of academic underperformance

**DOI:** 10.3402/meo.v19.22919

**Published:** 2014-04-01

**Authors:** Terry D. Stratton, Carol L. Elam

**Affiliations:** Department of Behavioral Science, Office of Medical Education, University of Kentucky College of Medicine, Lexington, KY, USA

**Keywords:** admissions, underperformance, selection, at-risk students

## Abstract

**Background:**

Despite medical school admission committees’ best efforts, a handful of seemingly capable students invariably struggle during their first year of study. Yet, even as entrance criteria continue to broaden beyond cognitive qualifications, attention inevitably reverts back to such factors when seeking to understand these phenomena. Using a host of applicant, admission, and post-admission variables, the purpose of this inductive study, then, was to identify a constellation of student characteristics that, taken collectively, would be predictive of students at-risk of underperforming during the first year of medical school. In it, we hypothesize that a wider range of factors than previously recognized could conceivably play roles in understanding why students experience academic problems early in the medical educational continuum.

**Methods:**

The study sample consisted of the five most recent matriculant cohorts from a large, southeastern medical school (n=537). Independent variables reflected: 1) *the personal demographics of applicants* (e.g., age, gender); 2) *academic criteria* (e.g., undergraduate grade point averages [GPA], medical college admission test); 3) *selection processes* (e.g., entrance track, interview scores, committee votes); and 4) *other indicators of personality and professionalism* (e.g., Mayer-Salovey-Caruso Emotional Intelligence Test™ emotional intelligence scores, NEO PI-R™ personality profiles, and appearances before the Professional Code Committee [PCC]). The dependent variable, first-year underperformance, was defined as ANY action (repeat, conditionally advance, or dismiss) by the college's Student Progress and Promotions Committee (SPPC) in response to predefined academic criteria. This study protocol was approved by the local medical institutional review board (IRB).

**Results:**

Of the 537 students comprising the study sample, 61 (11.4%) met the specified criterion for academic underperformance. Significantly increased academic risks were identified among students who 1) had lower mean undergraduate science GPAs (OR=0.24, *p*=0.001); 2) entered medical school via an accelerated BS/MD track (OR=16.15, *p*=0.002); 3) were 31 years of age or older (OR=14.76, *p*=0.005); and 4) were non-unanimous admission committee admits (OR=0.53, *p=*0.042). Two dimensions of the NEO PI-R™ personality inventory, openness (+) and conscientiousness (−), were modestly but significantly correlated with academic underperformance. Only for the latter, however, were mean scores found to differ significantly between academic performers and underperformers. Finally, appearing before the college's PCC (OR=4.21, *p=*0.056) fell just short of statistical significance.

**Conclusions:**

Our review of various correlates across the matriculation process highlights the heterogeneity of factors underlying students’ underperformance during the first year of medical school and challenges medical educators to understand the complexity of predicting who, among admitted matriculants, may be at future academic risk.

As the breadth of attributes and capabilities defining modern physicians has continued to expand, so too has the challenge of reliably assessing the future potential of medical school applicants. For many admission committees, this also entails gauging the appropriate ‘fit’ of applicants within a given programmatic focus or mission – balancing the demands of the profession with those of the school, the geographic region, or the local population (for example). Thus, a guiding principle of holistic review is the alignment of admission practices and the relative values of selection criteria with institutional missions and goals (1, 2). Indeed, Edwards and colleagues contend that medical schools should consider devising admission models to clarify the role and contribution of multiple components to the overall function of the admission process (3). In their view, an admission model consists of: 1) the applicant pool; 2) criteria for selection; 3) the admission committee; 4) selection processes and policies, and 5) outcomes (3).

Reviewing the function of each aforementioned component and exploring the many interrelationships illuminates the overall function of the admission process (1, 3, 4). Deconstructing this model highlights not only the size of the applicant pool but also the applicants’ diversity and sociodemographic characteristics. With regard to the admission criteria, we can assess and weigh cognitive variables such as college major, undergraduate grade point averages (GPAs), the Medical College Admission Test (MCAT) scores, and consider non-cognitive, personal qualities – including those gleaned via the medical school interview, letters of evaluation, personal statements, or psychological tools (5).

Similarly, consideration should be given to the backgrounds of the admission committee members and how those individuals may influence the deliberative and voting process in admission decision making (6–8). Selection practices may include such factors as screening processes, consideration given to students applying through special programs (e.g., early decision, combined degree programs), or the point in the admission cycle that an applicant receives notification of acceptance (e.g., during the regular admission period, later from the alternate list, etc.). Finally, both short-term (e.g., routine promotion during medical school, completion of USMLE Step Examinations, or appearances before professional code or student progress committees) and long-term outcomes (e.g., specialty selection, practice location, or state medical board disciplinary action) have the potential to inform aspects of each school's medical admission process (9).

Evaluating the effectiveness of admission policies, processes, and criteria in producing outcomes that reflect a medical school's mission is a core element of holistic review (1). That said, despite the considerable time and effort expended by admission committees to select the very best students from the growing pools of increasingly talented applicants, a number of students in any given cohort will invariably struggle academically during the first year of medical school. In some instances, vulnerabilities may be recognized – and deemed to be acceptable risks that are offset by other aptitudes, attributes, or backgrounds that applicants bring to their class, the program, or the profession. In other cases, however, classroom struggles inexplicably befall a small handful of students with no obvious predisposition to academic underperformance.

Such failures to perform satisfactorily in medical school could reflect problems within the admission process – or issues that might be addressable during the admission process or early in students’ matriculation (10, 11). Because the human and financial costs of medical students’ academic failure are high (12), it is incumbent upon medical school administrators and admission committee members in general, and admissions officers in particular, to undertake a careful review of each component of their admission model.

This study, then, uses a holistic review of our medical admission process by retrospectively examining academic underperformance during year one relative to variables associated with 1) *the applicant pool* (sociodemographic characteristics); 2) *selection criteria* (cognitive and non-cognitive factors); 3) *selection processes* (admission-related factors); and 4) *other factors of interest* – including personality measures and professionalism indicators that are not currently part of the admission process.

## Method

The study sample consisted of matriculants from the University of Kentucky College of Medicine's (UKCOM) classes of 2009–2013 (*n*=537). From multiple sources, a database was assembled which consisted of variables linked to 1) *the personal demographics of applicants* (age, gender, race, resident or non-resident, and Kentucky county of origin [rural, rural Appalachian, urban, Urban Appalachian]); 2) *academic criteria* (undergraduate college major, undergraduate college school, undergraduate major, undergraduate college science, non-science, and total GPA and MCAT subscale scores); 3) *selection processes* (entrance track [BS/MD, MD/MPH, MD/PhD, regular MD], early decision status, interview scores, committee voting patterns, and regular vs. alternate acceptances); and 4) *other indicators of personality and professionalism* (scores on the Mayer-Salovey-Caruso Emotional Intelligence Test [MSCEIT™], scores on the Revised NEO Personality Inventory [NEO PI-R™], and administrative records of student appearances before the UKCOM Professional Code Committee [PCC]) (13, 14).

The dependent variable, a short-term outcome, was underperformance during students’ M1 year – as defined by ANY *action* (repeat, conditional advancement, dismissal) by the college's Student Progress and Promotion Committee (SPPC) in response to the following academic criteria: 1) GPA≤2.5 or deficiency in any course; or 2) GPA≤2.0, 2, or more ‘E’ grades, OR 3 or more ‘U’ grades. (Both ‘E’ and ‘U’ grades reflect unsatisfactory academic performance. However, while the former are permanent, the latter are temporary and reflect a deficiency that might, given available evidence, be remediated upon completion of make-up work.)

SPSS (Version 22.0) was used for all analyses – with alpha specified as <0.05 for all interferential statistical tests (15). This study protocol was approved by the University of Kentucky Medical Institutional Review Board (IRB).

## Results

### Bivariate analyses

Of the 537 students comprising the study group, 61 (11.4%) met the specified criterion as underperforming during their M1 year. Of those, 32 were promoted to second year, 26 were required to repeat first year, and 3 were dismissed from the program. Since data for selected variables (e.g., personality indicators) were not available for all students, we chose not to sample from this population (as defined). As a result, study subjects represent an enumeration of the five academic cohorts, but with incomplete data on some measures.

Preliminary bivariate analyses revealed several statistically significant findings. First, students aged 31 years and older (*n*=25) were significantly more likely than their younger counterparts to academically underperform during their first year (*X* ^2^=13.31, *df*=3, *p*=0.004). Indeed, within this age group, nearly one student in three (32.0%) exhibited academic difficulties during Year 1.

Second, the largest proportion of academic underachievement was noted among African American students (*n*=27), where, again, nearly one student in three (29.6%) struggled academically during their first year (*X* ^2^=12.34, *df*=2, *p*=0.002). Third, students who appeared before SPPC were found to have significantly lower mean undergraduate (college) science (*t=*4.01, *df*=535, *p*≤0.001) and total (*t*=3.54, *df*=535, *p*≤0.001) GPAs. Average non-science GPA, while lower, was not statistically different.

Fourth, despite very small numbers (*n*=8), a Fisher's Exact test found that a larger but non-significant percentage of students who entered medical school early via the BS/MD track (37.5%) exhibited academic difficulties (*p*=0.052).

Fifth, among students for whom admission committee voting data were available (*n*=458), a significantly smaller percentage of those who garnered unanimous support were found to have first-year academic difficulties (*X* ^2^=6.01, *df*=1, *p*=0.014), compared with those for whom there was at least one dissenting vote. Interestingly, however, the level of dissent or disagreement among committee members was unrelated to academic underperformance, as defined.

Finally, based on the 314 students who completed the NEO PI-R™ during first-year orientation to medical school, moderate but statistically significant correlations were found between two dimensions and academic underperformance: Openness (*r*_*s*_=0.13, *p*=0.021) and conscientiousness (*r*_*s*_=−0.14, *p*=0.016). However, comparisons of mean scores found a difference only for the latter (*t*=2.23, *df*=307, *p*=0.024), with academic underperformers being significantly less conscientious. These same students, on average, were also higher on the ‘Openness’ dimension, although this difference was not statistically significant (*t*=−1.93, *df*=307, *p*=0.055). Based on the reduced subset (n=200) of students who completed the MSCEIT™, no dimension of emotional intelligence was significantly associated with academic problems in the first year. (Note: although response rates for the NEO PI-R and MSCEIT were quite high (>80%), these rather lengthy instruments were not administered to all student cohorts due to time constraints).

### Multivariate analyses

To accommodate a dichotomous dependent variable (appearance before the College's SPPC), binary logistic regression analyses were conducted using significant zero order correlates of academic underperformance as covariates. A forced entry protocol was used to enter individual covariates incrementally into the model to examine changes in the magnitude of effects on predictors. However, tabular results reflect only the final, cumulative effects of all variables in the model, rather than changes in effects at each step.

In contrast to linear regression, which estimates the individual and cumulative effects of predictors on a continuous dependent variable, logistic regression estimates (via the specified independent variables) the probabilities that a given observation belongs in each of two (binomial) or more (multinomial) groups. These probabilities are presented as odds – the natural logarithm of which (or logit) represents each regression coefficient (*β*). Thus, the binomial multivariate logistic regression prediction equation is:

$$\ln\left(\frac{\overset{⌢}{\rho }}{1-\overset{⌢}{\rho }}\right)=\beta_{0}+\beta_{1}X_{1}+\beta_{2}X_{2}+\ldots \ldots \beta_{k}X_{k}$$

A more interpretable means of conveying the strength of a relationship is the odds ratio, or exponentiated *β* [Exp(*β*)], which reflects changes in the odds of belonging to one group of the dependent variable for every one-unit increase in a given independent variable. Whereas probabilities, by definition, are bound between 0 and 1, odds (and odds ratios) have no upper limit, with an odds ratio [Exp(*β*)] of 1.0 representing an equal likelihood of belonging to either group.

The first model included all substantive covariates excluding admissions committee voting data (variable: ‘unanimous admit’), the two NEO PI-R™ dimensions: Openness and conscientiousness. Since these measures were not available for all students, omitting them from the initial analysis maximized the analyzable sample size – providing a more robust predictive model. Subsequently, these covariates were incrementally included in the second and final analysis – necessitating the reduction of cases in a list-wise fashion. These latter models, then, are based on more restricted (smaller) samples.

Table 1 shows the unique effects of each covariate after controlling for all other variables in the model. In particular, the odds of a student eliciting action from the college's SPPC were significantly greater for matriculants who 1) were 31 years of age or older (OR=7.96, *p*=0.022); 2) entered medical school via the accelerated BS/MD track (OR*=*7.76, *p*=0.014); 3) had lower mean undergraduate science GPAs (OR=0.41, *p*=0.018); and 4) had appeared before the college's PCC (OR*=*4.25, *p*=0.042). Students’ race, which initially showed a marked negative effect for African American students, was attenuated to non-significant levels after controlling for undergraduate science GPA.

**Table 1:** Logistic regression analysis of academic underperformance^a^ among undergraduate medical students (n=537)

Independent variable	*β*	SE *β*	Sig *β*	Exp(*β*) [95% CI]
Race^b^				
African American	0.98	0.51	0.056	2.66 [0.98, 7.25]
Other non-Whites	0.44	0.37	0.242	1.55 [0.74, 3.23]
Age^c^				
22–25	1.29	0.78	0.102	3.59 [0.78, 16.57]
26–30	1.06	0.88	0.228	2.89 [0.51, 16.17]
≥31	2.07	0.91	0.022	7.96 [1.35, 47.07]
BS/MD track^d^	2.05	0.83	0.014	7.76 [1.35, 39.60]
Undergraduate science GPA	−0.91	0.38	0.018	0.41 [0.19, 0.86]
Professionalism^e^	1.45	0.71	0.042	4.25 [1.05, 17.16]
Constant	−0.33	1.64	0.841	0.72
Model *X* ^2^=33.75, *df*=8, *p*≤0.001				

a Academic underperformance is coded ‘1’ for those who appeared before the Student Progress and Promotions Committee (SPPC) and ‘0’ for those who did not.

b Contrasts indicate the presence or absence of category membership. White (Caucasian) is the reference category.

c Contrasts indicate the presence or absence of category membership. Age (≤21) is the reference category.

d BS/MD track is coded ‘1’ for yes and ‘0’ for no. The latter is the reference category.

e Professionalism is coded ‘1’ for those who appeared before the Professional Code Committee (PCC) and ‘0’ for those who did not. The latter is the reference category.

In the second regression analysis, then, one new variable was introduced: whether or not the student was admitted unanimously by the admission committee. (Student's race, which remained a ‘borderline’ variable in Table 1, was initially retained but removed after its effects were shown to be further attenuated. Hence, it does not appear in subsequent analyses.) Since voting data were not available for all matriculants, the sample was slightly reduced to 458.

Table 2 shows the cumulative effects of this additional variable on the model predicting student underperformance. While the unique contributions of being a unanimous admit (or not) fell just short of the specified critical alpha (≤0.05), it also reduced the effects of the ‘professionalism’ variable. All other predictors, however, remained statistically significant.

**Table 2:** Logistic regression analysis of academic underperformance^a^ among undergraduate medical students (n=458)

Independent variable	*β*	SE *β*	Sig *β*	Exp(*β*) [95% CI]
Age^b^				
22–25	1.15	0.83	0.164	3.16 [0.62, 15.98]
26–30	0.70	0.94	0.458	2.01 [0.32, 12.85]
≥31	2.57	0.96	0.007	13.06 [2.00, 85.22]
BS/MD track^c^	2.80	0.89	0.002	16.48 [2.89, 93.97]
Undergraduate science GPA	−1.40	0.42	0.001	0.25 [0.11, 0.56]
Professionalism^d^	1.44	0.75	0.056	4.21 [0.96, 18.41]
Unanimous decision^e^	−0.61	0.31	0.051	0.54 [0.29, 1.00]
Constant	2.02	1.70	0.236	7.52
Model *X* ^*2*^=41.09, *df*=7, *p*≤0.001				

a Academic underperformance is coded ‘1’ for those who appeared before the Student Progress and Promotions Committee (SPPC) and ‘0’ for those who did not.

b Contrasts indicate the presence or absence of category membership. Age (≤21) is the reference category.

c BS/MD track is coded ‘1’ for yes and ‘0’ for no. The latter is the reference category.

d Professionalism is coded ‘1’ for those who appeared before the Professional Code Committee (PCC) and ‘0’ for those who did not. The latter is the reference category.

e Unanimous decision is coded ‘1’ for those who received all positive votes (to admit) from the admissions committee and ‘0’ for those who did not. The latter is the reference category.

Preliminary analyses sought to examine the potential effects of specific non-cognitive attributes, – namely emotional intelligence (MSCEIT™) and personality (NEO PI-R™), on student underperformance. Two dimensions of the NEO PI-R™ personality inventory (previously detailed) noted modest but statistically significant zero order correlations with first-year academic underperformance: openness (*r*_*s*_=0.13, *p*=0.02) and conscientiousness (*r*_*s*_=−0.14, *p=*0.02). However, since these data were available only for a small (*list-wise n*=265) subset of students, attempts to incorporate these additional variables into the previously specified model proved unsuccessful. As a result, we were unable to ascertain the effects of openness and conscientiousness relative to other predictors. No aspect of the MSCEIT™ was significantly associated with academic underperformance.

Table 3, then, contains final logistic regression analysis, which consists of four significant predictors of students’ academic underperformance during the first year of medical school: 1) Undergraduate science GPA; 2) BS/MD track of entry; 3) age (≥31 years of age); and 4) unanimous admission committee admit. The final equation predicting academic underperformance is:

$$\ln\left(\frac{\overset{⌢}{\rho }}{1-\overset{⌢}{\rho }}\right)=2.12-(1.41*\text{SciGPA})+(2.78*\text{BS}-\text{MD})+(2.69*\text{age})-(0.63*\text{unanimous})$$

**Table 3:** Logistic regression analysis of academic underperformance^a^ among undergraduate medical students (n=458)

Independent variable	*β*	SE *β*	Sig *β*	Exp(*β*) [95% CI]
Age^b^				
22–25	1.14	0.83	0.168	3.13 [0.62, 15.89]
26–30	0.78	0.94	0.408	2.17 [0.34, 13.69]
≥31	2.69	0.95	0.005	14.76 [2.29, 95.23]
BS/MD track^c^	2.78	0.89	0.002	16.15 [2.83, 92.16]
Undergraduate science GPA	−1.41	0.41	0.001	0.24 [0.11, 0.54]
Unanimous decision^d^	−0.63	0.31	0.042	0.53 [0.29, 0.98]
Constant	2.12	1.69	0.209	8.36
Model *X* ^*2*^=37.71, *df*=6, *p*≤0.001				

a Academic underperformance is coded ‘1’ for those who appeared before the Student Progress and Promotions Committee (SPPC) and ‘0’ for those who did not.

b Contrasts indicate the presence or absence of category membership. Age (≤21) is the reference category.

c BS/MD track is coded ‘1’ for yes and ‘0’ for no. The latter is the reference category.

d Unanimous decision is coded ‘1’ for those who received all positive votes (to admit) from the admissions committee and ‘0’ for those who did not. The latter is the reference category.

## Discussion

Using appearance before our college's progress and promotions committee as the dependent variable and short-term outcome, this study examined a multitude of factors relative to students’ first-year academic underperformance in order to evaluate components of our admission process using holistic review principles (1). Although a number of factors were established as correlates of academic underperformance, the results of this study did not identify problematic areas within our admission process.

Our analysis revealed that 11.4% (*n*=61) of students in our multi-year sample met this criterion for underperformance – a percentage comparable to the 14% figure cited by Durning and colleagues (16), who used a similar operational definition. Students’ age, undergraduate science GPA, entrance via an accelerated BS/MD track, and whether or not they were unanimous decisions for admission (by the medical school admission committee) were all significantly predictive of academic underperformance.

Interestingly, no aspect of the MCAT was found to be significantly associated with students’ academic underperformance. Instead, undergraduate science GPA showed a moderate and consistent negative relationship, with lower figures increasing the likelihood of a student's appearance before SPPC. One possible explanation may have to do with differences in the nature and structure of each indicator: While the MCAT is a cross-sectional, episodic assessment that can be prepared for (and taken repeatedly), undergraduate science GPA represents a longitudinal, continuous measure that may reflect other desired attributes, such as persistence, stamina, determination, conscientiousness, and so on.

The student age variable warrants further attention. Indeed, it is curious that both BS/MD track students, who tend to be comparatively *younger*, and *older* students in general were at significantly greater risk to appear before SPPC. It is quite possible, however, that different factors underlie each group. For example, age in younger students may be a proxy for maturity, while age in older students may reflect time away from full-time academics.

As a zero-order correlation, student race initially showed African American students to be at significantly greater risk for academic underperformance during the first year. However, this effect was attenuated to a non-significant level by undergraduate science GPA and, to a lesser extent, age. Other studies have found majority and minority students’ performance in medical school to be impacted by slightly different sets of factors (17). Unfortunately, our sample lacked the racial heterogeneity to fully explore this possibility.

To the extent that institutional missions dictate that admission committees tolerate a certain level of academic risk in order to matriculate well-qualified students from a spectrum of backgrounds, the potential to identify academically vulnerable applicants reliably is useful not so much in ‘screening out’ these individuals, but rather in directing to them the necessary resources to overcome anticipated obstacles.

Of course, identifying factors predictive of academic underperformance – and ‘at risk’ individuals – is a necessary but not sufficient response to balancing institutional missions with academic demands. Where targeted resources are available, we are further motivated to understand why some underperforming students either deny their predicament or grossly underestimate the level of corrective action – sometimes to the point of refusing assistance that is offered.

Although many schools have in place resources for preemptive academic remediation (18), they may rely too heavily on students’ abilities to self-assess their situation and purposively seek out assistance (19). In this regard, the roles of non-cognitive factors related to personality, attitudes, or other factors may moderate the manifestation of students’ academic woes and, in some cases, may even play a more significant role in medical school performance than the usual selection criteria. (For a comprehensive review of non-cognitive constructs, see Megginson (20)).

Although the various stakeholders vested in the selection and admission of medical students have been shown to prioritize similar values vis-à-vis applicant characteristics (6), different populations may well warrant the consideration of different non-academic variables (21). Similarly, the actual admission process by which schools triage and select applicants must also be considered in relation to other relevant factors (7).

The admission committee at our school has reviewed the results of this study and recognizes the need to examine more closely the entire ‘package’ and preparedness of students with lower-than-average undergraduate science GPAs – or older, ‘non-traditional’ applicants who are seeking to enter professional school after a hiatus from classroom instruction. Toward this end, committee members now expect the former to have done graduate work or demonstrated competitive MCAT scores, while the latter are strengthened by any recent completion of full-time coursework prior to applying to medical school. Similarly, motivation for careers in medicine and life priorities is considered especially pertinent for ‘non-traditional’ applicants, both of which are carefully explored vis-à-vis the written application and face-to-face interview.

The admission processes for the BS/MD program have also been refined, with a clearer focus on maturity levels and study skills. Moreover, a renewed emphasis has been placed on monitoring the personal and academic progress of students during the baccalaureate portion of the program. Finally, the contribution of ‘conscientiousness’ to early academic success – while intriguing – remains relatively unaddressed. At this time, there are no plans to introduce completion of the NEO PI-R to the selection process for our medical students.

Several study limitations should be noted. First, we focused on one aspect of medical student success: academic achievement during the first year – as measured by students’ appearance before our college's progress and promotion committee. While specifying cumulative first-year GPA as our dependent variable may have resulted in some slight variations, our operational definition paralleled that of Durning and associates (16). Similarly, students’ future academic performance tends to be strongly predicated on their previous academic performance (17) – although this relationship is, admittedly, far from perfect.

Second, our use of non-cognitive measures, a class of constructs garnering increasing attention by numerous stakeholders, was necessarily selective. While the MSCEIT™ and NEO PI-R™ reflect commonly used measures of emotional intelligence and personality, respectively, they constitute only a sampling of what may be relevant and measureable (20). Moreover, they are not part of the admission process at our institution. On a more tangible level, data from the MSCEIT™ and NEO PI-R™ were available only for a subset of students (*n*=200 and 314, respectively), limiting our ability to examine their relationships to academic underperformance in a more comprehensive context.

Finally, in collapsing the continuous but positively skewed age variable, we chose the designated groupings based on the ranges of students who commonly enter medical school: 1) as part of the BS/MD program (≤21 years); 2) directly from college (22–25 years); 3) after addressing application deficiencies – or gaining life experiences/additional education (26–30 years); and 4) following career changes (≥31 years). While far from arbitrary, it is possible that an alternative categorization scheme may have resulted in different findings.

Future research may wish to explore related questions: What is not currently being measured that is important to understanding academic success? What interventions might be developed to identify and offset potential academic difficulties? What factors inhibit or dissuade at-risk students from seeking help or accepting remediation? Only by further elaborating the complex model of student success will the notion of a holistic admission process be fully realized. It is our hope that the process demonstrated here will reinforce the merits of a holistic approach when reviewing admission-related components vis-à-vis student progress by broadening the context beyond admission to include school-specific, personal, or other environmental factors.
